# Mitochondrial dynamics and the cell cycle

**DOI:** 10.3389/fpls.2014.00222

**Published:** 2014-05-27

**Authors:** Penny M. A. Kianian, Shahryar F. Kianian

**Affiliations:** ^1^Department of Horticultural Science, University of MinnesotaSt. Paul, MN, USA; ^2^Cereal Disease Laboratory, United States Department of Agriculture – Agricultural Research ServiceSt. Paul, MN, USA

**Keywords:** nuclear-mitochondrial communication, mitochondrial dynamics, cell cycle, mitosis, meiosis, alloplasmic

## Abstract

Nuclear-mitochondrial (NM) communication impacts many aspects of plant development including vigor, sterility, and viability. Dynamic changes in mitochondrial number, shape, size, and cellular location takes place during the cell cycle possibly impacting the process itself and leading to distribution of this organelle into daughter cells. The genes that underlie these changes are beginning to be identified in model plants such as *Arabidopsis*. In animals disruption of the *drp1* gene, a homolog to the plant *drp3A* and *drp3B*, delays mitochondrial division. This mutation results in increased aneuploidy due to chromosome mis-segregation. It remains to be discovered if a similar outcome is observed in plants. Alloplasmic lines provide an opportunity to understand the communication between the cytoplasmic organelles and the nucleus. Examples of studies in these lines, especially from the extensive collection in wheat, point to the role of mitochondria in chromosome movement, pollen fertility and other aspects of development.

Nuclear-mitochondrial (NM) communication impacts all aspects of plant development including fertility. The best known case of incompatibility or NM miscommunication is cytoplasmic male sterility (CMS, reviewed in Chase, 2006) and can result from the disruption of energy production (Pla et al., 1995; Gutierres et al., 1997; Brangeon et al., 2000; Sabar et al., 2000; Bergman et al., 2000; Ducos et al., 2001; Dieterich et al., 2003; Yui et al., 2003; Kim and Kim, 2006; León et al., 2007). Incompatibility can also be seen between the mitochondria and the nucleus in alloplasmic lines, created through the exchange of cytoplasmic organelles while maintaining the nuclear genome (Tsunewaki, 1980; Maan, 1987). Other phenotypic variations also are observed in these alloplasmic lines due to mitochondria’s role as the powerhouse of the cell. NM communication and the underlying molecular mechanism is not well understood, but is an area of developing research in plants (reviewed in Schwarzländer and Finkemeier, 2013). A critical area of research in this regard is the timing and control mechanisms for equitable distribution of chromosomes and organelles to new daughter cells after cell division. This review focuses on mitochondria fusion and fission during the cell cycle of plants, and its connection to chromosome movement.

## PLANT GENES INVOLVED IN MITOCHONDRIA FISSION AND FUSION

Throughout the plant’s cell cycle, mitochondria undergo changes in number, shape and location. These dynamic changes in the mitochondria are only beginning to be understood in plants compared to a greater understanding in yeast and animals (reviewed in Gorsich and Shaw, 2004; Hales, 2010; Westermann, 2010; Chan, 2012). *Arabidopsis* has been the model plant species for understanding the genes underlying the dynamic changes in mitochondria. There are several genes identified affecting mitochondria fusion and division (reviewed in Scott and Logan, 2011). Mutations in these nuclear encoded genes result in larger size, decreased number, altered shape, or mitochondrial network formation. Several of these genes were identified using ethyl methane sulphonate (EMS) mutagenesis. These mutants include the big mitochondrial mutant (*BMT1*), network mitochondrial mutant (*NETWORK1*), friendly mitochondrial mutant (*FMT*), and motley mitochondrial mutant (*MMT1*, *MMT2;* Logan et al., 2003). None of these mutants have any reported orthologs in yeast or humans. Other mitochondrial mutants affecting dynamics do have orthologs in yeast or humans. The genes *BIGYIN1* and *BIGYIN2* are orthologous to *FIS1* and *FZOI* (Scott et al., 2006; Lingard et al., 2008; Zhang and Hu, 2008; Scott and Logan, 2011). The remaining genes, *DRP3A* and *DRP3B,* are dynamin-related proteins with a role in mitochondrial fission (Arimura and Tsutsumi, 2002; Arimura et al., 2004; Logan et al., 2004). These are orthologous to the yeast and human DRP1. In *Arabidopsis*, it is speculated the *BIGYIN* genes may interact with dynamins such as *DRP3A* or *DRP3B* during mitochondria division, but experimental evidence for this hypothesis is lacking (Scott and Logan, 2011).

An understanding of how these genes interface with the cell cycle, and if their homologs exist in other plant species will be necessary to improve our understanding of NM interactions and their role in mitochondrial dynamics during the cell division process.

## MITOCHONDRIA FUSION AND FISSION DURING MITOSIS AND MEIOSIS

Understanding the timing and mechanism of mitochondrial fusion, and fission during the cell cycle is critical to determining their role in plant development. Using a limited number of plant species and experimental systems these changes are beginning to be appreciated. Mitochondrial movement in mitosis is mediated by the actin cytoskeleton fibers. The movement of the mitochondria is at a rate of 10 μm/s (Sheahan et al., 2004). There are no reports of how the mitochondria move during meiosis in plants, but it is expected to be similar.

In addition to movement, there is also a change in the number and shape of the mitochondria during mitosis. In tobacco protoplast cells, mitochondria undergo fusion to develop a tubular structure (Sheahan et al., 2004). At 24 h into the cell cycle, there is a decrease in the total number of mitochondria, per protoplast but the net cellular content remains the same. After the formation of the tubular structure, fission is predominantly observed within these cells resulting in small mitochondria. A high density of mitochondria is observed along the newly formed cell wall. At 72 h, the process of mitosis is complete. The number of mitochondria is doubled and dispersed between the two cells (Sheahan et al., 2004).

Like the tobacco protoplast, there are mitochondrial changes in *Arabidopsis* during the cell cycle. During the G1/S stages of the cell cycle in the shoot apical meristem, there is one large mitochondria found at the end of the nucleus with additional small round shaped mitochondria in other parts of the cell. During the G2, the large and small mitochondria double in size. At the M phase, about 60% of the small mitochondria will fuse with the large mitochondria in the cell. The large mitochondria surrounding the nucleus during the M phase has been described as a “cage.” It is hypothesized the “cage” formation allows for the mixing of mitochondria contents, including DNA, before distribution to the daughter cells (Seguí-Simarro and Staehelin, 2009). The large “cage” mitochondria will then divide, forming smaller rounded mitochondria, and re-distribute to the new cells similar to what is observed in tobacco protoplast cells. It is suspected that the large mitochondria provides the energy needed by the cell during the process of division (Seguí-Simarro et al., 2008). In the root-tip apical meristem, the presence of a single large mitochondria is not seen, the mitochondria maintain their classical rounded shaped (Seguí-Simarro et al., 2008). This difference in mitochondrial behavior in *Arabidopsis* between the two actively growing tissues suggests that there may be multiple distribution routes for mitochondria within a plant during the cell division.

During the process of meiosis there also are changes in plant mitochondria size and number. Mitochondrial changes, during meiosis, have been primarily observed during pollen development. Research in lily (*Lillium*) found that in the zygotene stage of prophase, mitochondria of the pollen mother cell begin to condense. They reach a diameter of 0.5 μm by leptonema, and would remain condensed until the tetrad stage (Bird et al., 1983). At the tetrad stage an increase in the number of mitochondria was found. Upon separation of the tetrads, the mitochondria return to their pre-meiotic state. Unfortunately, it is not clear when the number of mitochondria increases during meiosis. In barley pollen, the number of mitochondria was found to decrease as it matured. Immature pollen had a mean number of 62.0 mitochondria per pollen grain with an average size of 0.038 μM^3^, whereas mature pollen had a mean of 30.8 mitochondria per pollen grain with a size of 0.020 μM^3^ (Mogenson and Rusche, 1985). In maize pollen cells and protoplast from pollen, mitochondria were found as large, complex branching shapes with interconnections between the branches (McConchie et al., 1987; Wagner et al., 1988; Mogensen et al., 1990). These large mitochondria are reportedly positioned near the nucleus (McConchie et al., 1987; Wagner et al., 1988). It was noted that vegetative tissue surrounding the pollen did not have the same mitochondrial structure as found in the pollen (Mogensen et al., 1990). The mean number of mitochondria found was 43.4 per cell with a range of 7–74, and the average size of 3.97 μM^3^ (Mogensen et al., 1990). Comparing barley mature pollen to maize pollen protoplast, the number of mitochondria per cell is similar, but there is a difference in size by a factor of 200. These studies illustrate that mitochondria number and size change during pollen development. However, there is no unifying pattern of mitochondria fusion and fission found for plants species studied to date.

Similar to pollen development there are changes in mitochondrial number, size, and shape during megaspore development representing female gamete production in plants. The early stages of megasporogenesis in higher plants have similarity to the gametic development in female animals, with one cell developing into the egg and the remaining three haploid cells degrading. It may be expected to see a similar pattern of mitochondrial distribution, size and number in the plant megaspore as compared to animals.

Recent studies of mouse oocytes have revealed changes in mitochondria. There is an increase in the amount of mitochondrial DNA during meiosis I (Mahrous et al., 2012). In these oocytes, mitochondrial numbers reach over 100,000 per cell before the completion of meiosis II. At anaphase I when the homologous chromosomes separate, there is a biased inheritance in the number of mitochondria in the oocyte compared to the polar body. Estimates of the area occupied by mitochondria is about 23% in the oocyte compared to 4.8% for polar body (Dalton and Carroll, 2013). The number and biased inheritance of mitochondria in the mouse oocyte shows the importance of this organelle and the need for higher energy levels in animal gametes. Currently there is no information on the mitochondria’s role for energy production during female gametic development in plants.

Later stages of megaspore development differ between higher plants and animals. In animals, the mitochondria must produce the energy needed by the dividing cells to maintain the blastocyst until the embryo stage, when further mitochondria synthesis takes place (reviewed in Chappel, 2013). In plants, the megaspore nucleus will go through subsequent divisions without cytokinesis within the female gametophyte normally composed of the eight haploid nuclei (egg, 2-synergids, 3-antipodal, 2-polar). After the development of the eight haploid nuclei, the embryo sac is cellularized separating the cytoplasm and diluting the mitochondria between the cells (reviewed in Yang et al., 2010). The antipodal cells are thought to have a role in providing nutrients from the tissue surrounding the female gametophyte (Raghavan, 1997). The antipodal cells mitochondria could lower energy production demands on the embryo sac if they are also able to transfer energy along with the nutrients.

The female megaspore mitochondria number, location and shape have been analyzed in few plant species. In maize protoplast developed from egg cells, the mitochondria are found to be of various shapes including interconnected networks, and located near the nucleus of the egg (Faure et al., 1992). Similar to maize, a large filamentous mitochondria also is present in the *Capsella* embryo near the time of fertilization (Schulz and Jensen, 1973). This network will then disappears after the first round of endosperm division in *Capsella* (Schulz and Jensen, 1973). Instead of a branching network as seen in maize and *Capsella*, a large stacked collection of mitochondria are observed in the embryo sac of *Pelargonium zonale* (Kuroiwa et al., 1996). Whereas in the *Arabidopsis* egg cell, the mitochondria are described as rod or spherical shaped with a large number in the chalazal region (Yamaoka et al., 2011). There is also a substantial increase in the amount of mitochondrial DNA present during embryo sac development mirroring that reported in mouse oocytes. During the progression from immature to mature embryo sac in *P. zonale*, over a 900 fold increase in the amount of mitochondrial DNA is observed (Kuroiwa et al., 1996). This increase is mirrored in mouse oocytes, but estimates place the increase to be only 100-fold (Mahrous et al., 2012). These studies of pollen and megaspore development show that the mitochondria change shape, size, and cellular location during meiosis; yet linking these observations to the nuclear genes identified in *Arabidopsis* with a role in mitochondrial dynamics has not been established.

## MITOCHONDRIA AND CHROMATIN MOVEMENT DURING MEIOSIS

Research is ongoing to understand the effect of the mitochondria on chromosome movement. Humans have served as the model to understand this connection due to the decrease in fertility and increased frequency of aneuploidy with age (Schon et al., 2000; Bartmann et al., 2004; Eichenlaub-Ritter, 2012). Research in mice shows a disruption in energy production in the mitochondria was associated with an increase in diploid gametes. The diploid gametes were caused by lack of chromosome separation during meiosis. (Beermann and Hansmann, 1986). It is hypothesized, as humans age their mitochondria accumulates mutations. These mutations affect the organelle’s ability to adequately provide energy needed by the cell to successfully separate and move chromosomes during meiosis. The mis-segregation of chromosomes led to decreased fertility.

In plants, mutations in either nuclear or mitochondrial genes can result in sterility. Many of these identified mutations are associated with energy production in the mitochondria and are classified as CMS (Pla et al., 1995; Gutierres et al., 1997; Brangeon et al., 2000; Sabar et al., 2000; Bergman et al., 2000; Ducos et al., 2001; Yui et al., 2003; Kim and Kim, 2006; León et al., 2007). This disruption can affect the electron transport chain components including complex I and II, cytochrome C oxidase or the generation of ATP through mutations in the ATPase subunits. These studied CMS material have not been linked to changes in chromosome movement during meiosis or mitosis. However, mutations in mitochondrial genome alone may not be the only factor leading to aneuploidy in human cells. The process of fission and fusion of mitochondria also affects chromosome movement. The suppression of the nuclear gene *drp1*, with a role in mitochondrial fission in human cells, leads to cell cycle arrest at G_2_/M phase resulting in aneuploidy. Large mitochondrial networks remained in the *drp1* lines throughout the cell cycle as compared to the wild-type control with normal mitochondrial division. The delay in mitochondrial fragmentation correlated with an increased in chromosome mis-segregation. Researchers were able to uncouple mitochondrial dynamics from oxidative stress and energy production. This uncoupling illustrated that changes in fission can led to aneuploidy independent of energy levels (Qian et al., 2012). Within *Arabidopsis*, orthologs to *DRP1* have been identified. These are *DRP3A* and *DRP3B*, formerly ADL2b and ADL2a (Arimura and Tsutsumi, 2002; Arimura et al., 2004; Logan et al., 2004). These proteins linked to green fluorescent protein (GFP) tag have been shown to locate in the mitochondria near the point of division. Transient expression of either of the mutated proteins in tobacco cells resulted in larger mitochondria (Arimura and Tsutsumi, 2002; Arimura et al., 2004; Logan et al., 2004). It will be exciting to discover if *DRP3A* or *DRP3B* can affect chromosome movement during either mitosis or meiosis in plants similar to the observations in human cells.

## NUCLEAR-CYTOPLASMIC INTERACTIONS BEYOND CYTOPLASMIC MALE STERILITY

To understand the connection between the mitochondria and chromosome movement during the cell cycle, it will be necessary to understand the intricacies of nuclear-cytoplasmic (NC) interactions. NC interactions can have multiple effects on plants growth and development. CMS is the most studied interaction due to its importance to crop production (reviewed in Ghavami et al., 2014). However, the communication between the nucleus and cytoplasm can also impact aspects of plant growth including height, vigor, endosperm quality, and other traits.

**FIGURE 1:**
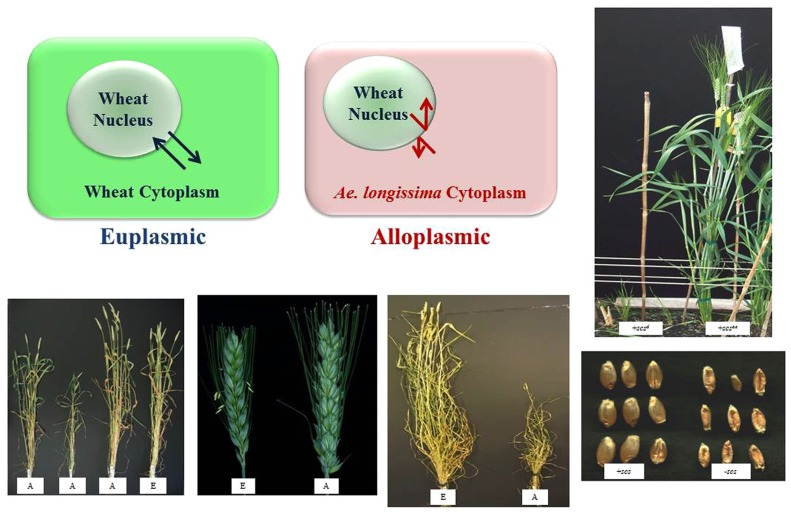
**Examples of euplasmic and alloplasmic wheat lines.** Top part of the figure is a diagrammatic representation of a euplasmic (true cytoplasm) and alloplasmic (alien cytoplasm, e.g., *Aegilops longissima*) lines of wheat. Euplasmic communication can take place between nucleus and cytoplasm as indicated by the arrows. Alloplasmic communication between nucleus and cytoplasm can be disrupted as indicated by opposing arrows. Bottom part of the figure provides examples of alloplasmic plants and the influence of cytoplasm on development. The first and third pictures are examples of cytoplasmic influence on vigor, middle figure is the influence on fertility and the two right most figures (both top and bottom) illustrate the influence of a single nuclear locus (*species cytoplasm specific* or *scs*) on seed and plant development in alloplasmic condition. In all cases A = alloplasmic and E = euplasmic.

Alloplasmic lines provide an opportunity to understand the communication taking place between the cytoplasmic organelles and the nucleus. In this material, the nucleus of one species is replaced with a nucleus of another while retaining the cytoplasmic genomes. This replacement can lead to changes in phenotype including sterility, vigor and viability (**Figure 1**; Tsunewaki, 1980; Ogihara et al., 1997, 1999; Bergman et al., 2000; Tsunewaki et al., 2002; Dieterich et al., 2003; Banga et al., 2003; Shinada et al., 2006; Vu et al., 2011). For instance, a line with *Triticum crassa* cytoplasm exhibits a photoperiod-sensitive homeotic transformation of anthers (Ogihara et al., 1997, 1999). It was determined that a number of alterations in mitochondrial DNA structure and transcription, as well as a nuclear MADS box gene, were responsible for this transformation (Murai et al., 2002; Yamamoto et al., 2013). This case illustrates the complexity of biochemical processes involved in NM interaction in alloplasmic wheat, where the phenotype is not a straightforward male sterility. In a male sterile tobacco alloplasmic line a decrease in the ATP + ADP pool of 55% in floral buds was found (Bergman et al., 2000). Further investigation of this line identified changes in the mitochondrial genome as compared to the euplasmic (wild type) control. The mitochondria in the alloplasmic line were larger and the intermembrane space appeared more “open” compared to the wild type (Farbos et al., 2001). It was hypothesized that decreased energy production in the alloplasmic line delayed developmental timing leading to sterility (Farbos et al., 2001). These examples are evidence of disruption in NM interactions changing plant phenotypes beyond the typically studied CMS phenotype.

Thus, analysis of genes involved in NM interaction is critical to understanding of the gene networks controlling the production of functional gametes and development of viable plants.

## CONCLUSION

Nuclear-mitochondrial interactions and the underlying molecular mechanism(s) are not well understood, but is an area of developing research in plants. Several genes have been identified in *Arabidopsis* with effects on mitochondrial size, shape, and number. How these genes are related to the changes observed in plant mitochondria during mitosis, pollen and female gametophyte development is not known. The importance of the mitochondria to cellular energy has been well studied. Yet the connection of mitochondria and its role to chromosome movement as seen in animals has not been established in plants. Knowledge of NM interactions will be beneficial to understanding mitochondrial dynamics, and chromosome movement.

## AUTHOR CONTRIBUTIONS

Penny M. A. Kianian and Shahryar F. Kianian contributed to the conception, drafting, publication, and are accountable for all aspects of this work.

## Conflict of Interest Statement

The author declares that the research was conducted in the absence of any commercial or financial relationships that could be construed as a potential conflict of interest.
